# Supplementary material for: A Simple, Cost-Effective, and Automation-Friendly Direct PCR Approach for Bacterial Community Analysis
Source: mSystems. 2021 Sep 28;6(5):e00224-21. doi: 10.1128/mSystems.00224-21 (PMC8547444; doi:10.1128/mSystems.00224-21)
Supplement: TABLE S3 [file msystems.00224-21-st003.pdf]

Reagents/kits	Brand (catelog number)	Volume/capacity	Price(USD)[a]	Usage Concentration	Reaction Volume	Total Volume of Reaction	Quantity of Reaction	Cost/ 96 samples (USD)	96 well plate included?	PCR reagents included?	Lysis cost/ 96 samples[c]
IGEPAL CA-630	Sigma-Aldrich (I8896-100ML)	100 ml (100%)	83.8	0.1%	50 ul	100 L	2X10^6	0.004	No	No	0.004
				0.5%	20 ul	20 L	1X10^6	0.008			0.008
Proteinase K	Qiagen (19133)	10 ml (20 mg/ml)	346	100 ug/ml	20 ul	2 L	1X10^5	0.33	No	No	0.33
DNeasy PowerSoil HTP 96 kit	Qiagen (12955-4)	384	2230	n.a	n.a	n.a	384	557.5	Yes (~$4.8 per plate[b])	No	552.7
Extract-N-Amp Plant PCR kit	Sigma-Aldrich (XNAR-1KT)	1000	2530	n.a	n.a	n.a	1000	242.9	No	Yes (~$66 per 96 reactions[b])	176.9

[a] List prices on their official websites.

[b] Cost of 96 well plate is estimated by Corning 96 well plates; and cost of PCR reagent is estimated by KAPA HiFi HotStart ReadyMix.

[c] Cost does not include PCR reagents (subtracted $66 per 96 samples from Extract-N-Amp kit) and consumables- 96 well plate (subtracted $4.8 per 96 samples from PowerSoil kit).
