# Supplementary figures and images for: A Simple, Cost-Effective, and Automation-Friendly Direct PCR Approach for Bacterial Community Analysis
Source: mSystems. 2021 Sep 28;6(5):e00224-21. doi: 10.1128/mSystems.00224-21 (PMC8547444; doi:10.1128/mSystems.00224-21)

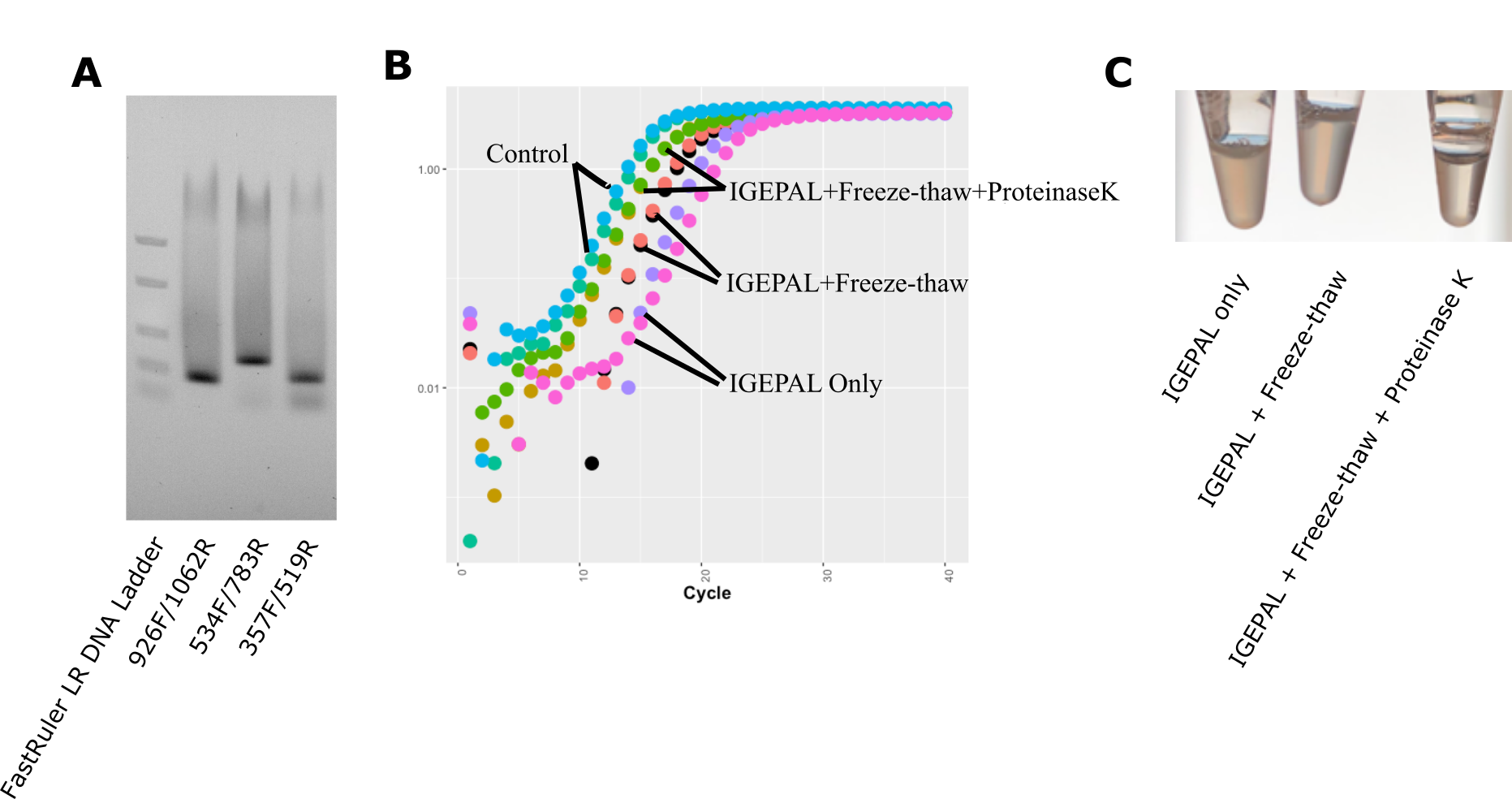

Supplement: FIG S1 [file msystems.00224-21-sf001.tif]

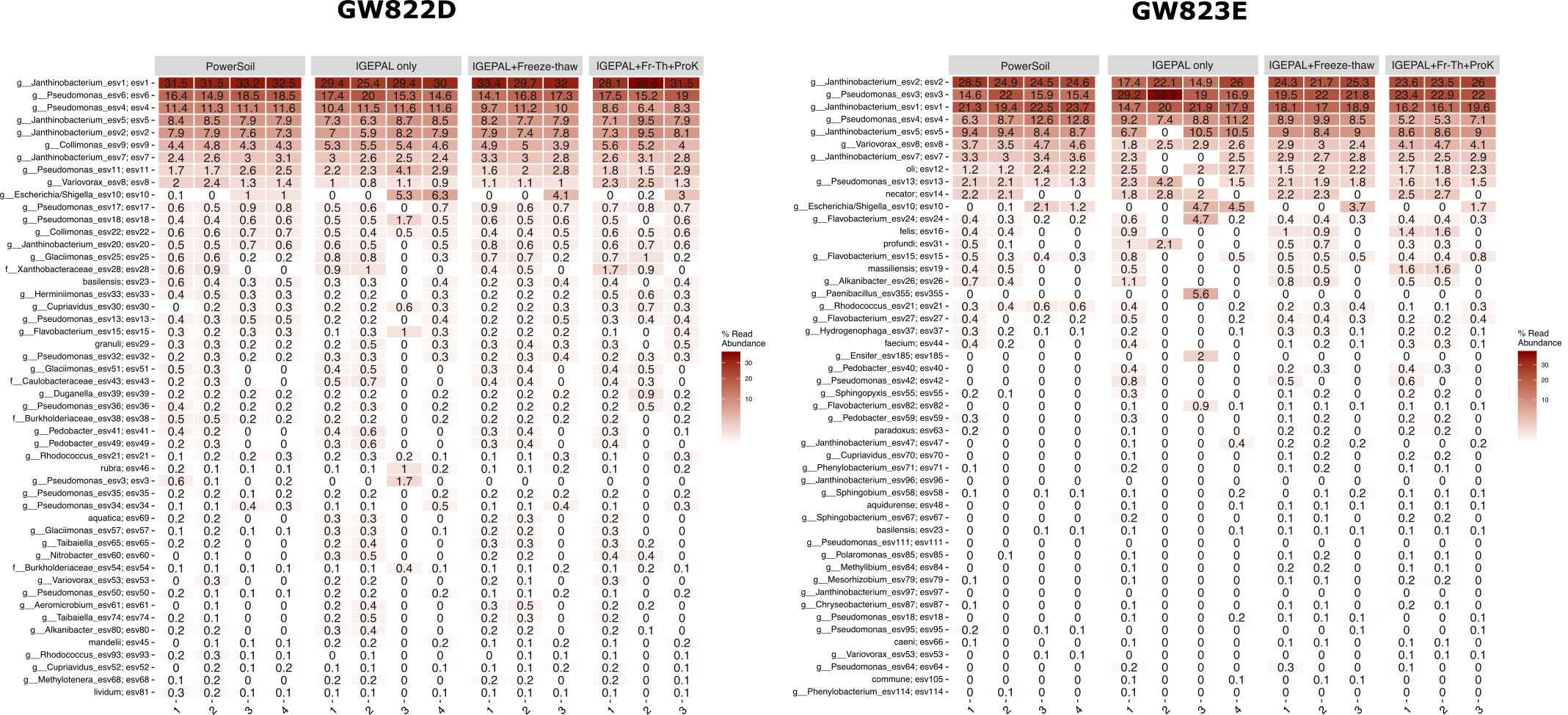

Supplement: FIG S2 [file msystems.00224-21-sf002.tif]

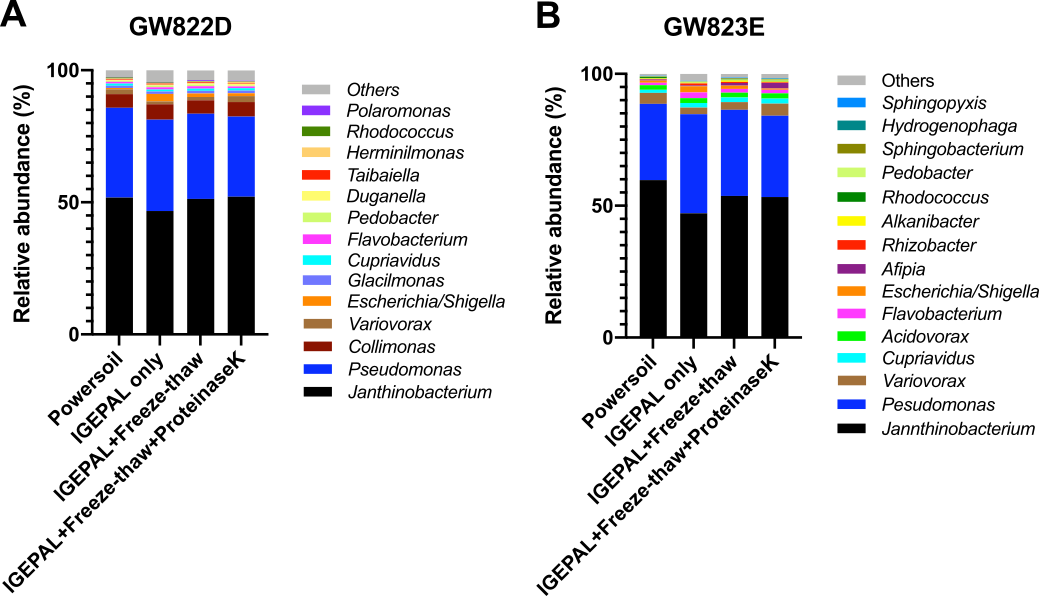

Supplement: FIG S3 [file msystems.00224-21-sf003.tif]

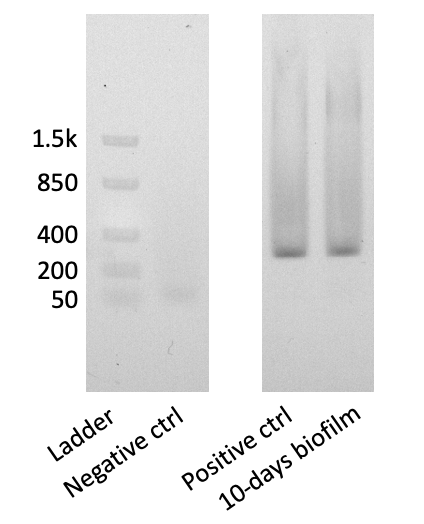

Supplement: FIG S4 [file msystems.00224-21-sf004.tif]
