# Supplementary material for: A Simple, Cost-Effective, and Automation-Friendly Direct PCR Approach for Bacterial Community Analysis
Source: mSystems. 2021 Sep 28;6(5):e00224-21. doi: 10.1128/mSystems.00224-21 (PMC8547444; doi:10.1128/mSystems.00224-21)
Supplement: TABLE S1 [file msystems.00224-21-st001.pdf]

		Escherichia Exponential	Escherichia Stationary	Pseudomonas Exponential	Pseudomonas Stationary	Lactococcus Exponential	Lactococcus Stationary	Lactobacillus Exponential	Lactobacillus Stationary
IGEPAL only	P value	0.3985	0.4587	0.1215	0.8627	<0.0001	<0.0001	<0.0001	<0.0001
	ΔCt	-1.733 ± 1.835	0.4938 ± 0.6028	-3.568 ± 1.820	0.06502 ± 0.3526	6.272 ± 1.127	2.409 ± 0.4388	3.931 ± 0.1825	30.18 ± 0.4252
IGEPAL+Freeze-thaw	P value	0.1918	0.1602	0.9065	0.4247	0.0157	<0.0001	<0.0001	<0.0001
	ΔCt	2.147 ± 1.368	0.4815 ± 0.3111	0.1567 ± 1.253	-0.4500 ± 0.5067	2.562 ± 0.8627	3.244 ± 0.4509	3.113 ± 0.2443	26.87 ± 0.5764
IGEPAL+Freeze-thaw+ProteinaseK	P value	<0.0001	0.0012	0.0014	<0.0001	0.787	<0.0001	0.1934	<0.0001
	ΔCt	-3.149 ± 0.2015	-1.748 ± 0.2626	-4.386 ± 0.6922	-2.178 ± 0.1866	0.1947 ± 0.6993	7.884 ± 0.5280	0.7051 ± 0.5016	12.86 ± 0.3844
