# Supplementary material for: A Simple, Cost-Effective, and Automation-Friendly Direct PCR Approach for Bacterial Community Analysis
Source: mSystems. 2021 Sep 28;6(5):e00224-21. doi: 10.1128/mSystems.00224-21 (PMC8547444; doi:10.1128/mSystems.00224-21)
Supplement: TABLE S2 [file msystems.00224-21-st002.pdf]

Relative abundance (%)	Lactobacilus fermentum	Enterococcus faecalis	Escherichia coli	Salmonella enterica	Pseudomonas aeruginosa	Staphylococcus aureus	Bacilus subtilis	Listeria monocytogenes
Control	18.4	9.9	10.1	10.4	4.2	15.5	17.4	14.1
PowerSoil_1	17.89	12.6	21.54	25.51	8.94	6.91	4.27	2.34
PowerSoil_2	15.42	16.52	17.82	13.41	10.01	14.31	8.21	4.3
PowerSoil_3	14.99	15.68	17.68	14.29	9.29	13.59	8.99	5.49
PowerSoil_4	21.02	9.81	21.92	21.62	9.01	7.21	6.81	2.6
PowerSoil_5	13.94	11.09	23.8	24.21	9.87	7.43	7.12	2.54
PowerSoil_6	11.22	15.13	19.54	16.63	10.82	12.32	9.62	4.71
IGEPAL only_1	67.9	14.1	5.3	2.1	1	6	1.4	2.2
IGEPAL only_2	60.32	16.93	5.91	2.81	1.7	8.42	1.9	2
IGEPAL only_3	65.53	15.13	5.31	2.51	1.3	6.81	1.7	1.7
IGEPAL+Freeze-thaw_1	55.26	17.82	6.81	3.4	1.8	9.71	2.8	2.4
IGEPAL+Freeze-thaw_2	51.9	20.04	7.92	3.81	2	9.62	2.2	2.51
IGEPAL+Freeze-thaw_3	52.05	20.22	7.61	3.7	1.9	9.71	2.3	2.5
IGEPAL+Freeze-thaw+ProteinaseK_1	11.72	32.67	17.84	9.52	4.01	13.73	4.81	5.71
IGEPAL+Freeze-thaw+ProteinaseK_2	13.3	33.5	18.5	9.9	3.3	11.9	4.4	5.2
IGEPAL+Freeze-thaw+ProteinaseK_3	16.02	32.73	16.02	9.01	2.9	12.21	5.41	5.71
